# RELAY: Robotic EyeLink AnalYsis of the EyeLink 1000 Using an Artificial Eye

**DOI:** 10.3390/vision9010018

**Published:** 2025-03-01

**Authors:** Anna-Maria Felßberg, Dominykas Strazdas

**Affiliations:** 1 Medical Faculty, Otto-von-Guericke University Magdeburg, 39120 Magdeburg, Germany; 2 Neuro-Information Technology, Otto-von-Guericke University Magdeburg, 39106 Magdeburg, Germany; dominykas.strazdas@ovgu.de

**Keywords:** accuracy, artificial eye, brightness, eye tracking, gaze, P-CR, peak velocity, precision, saccades, EyeLink 1000

## Abstract

The impact of ambient brightness surroundings on the peak velocities of visually guided saccades remains a topic of debate in the field of eye-tracking research. While some studies suggest that saccades in darkness are slower than in light, others question this finding, citing inconsistencies influenced by factors such as pupil deformation during saccades, gaze position, or the measurement technique itself. To investigate these, we developed RELAY (Robotic EyeLink AnalYsis), a low-cost, stepper motor-driven artificial eye capable of simulating human saccades with controlled pupil, gaze directions, and brightness. Using the EyeLink 1000, a widely employed eye tracker, we assessed accuracy and precision across three illumination settings. Our results confirm the reliability of the EyeLink 1000, demonstrating no artifacts in pupil-based eye tracking related to brightness variations. This suggests that previously observed changes in peak velocities with varying brightness are likely due to human factors, warranting further investigation. However, we observed systematic deviations in measured pupil size depending on gaze direction. These findings emphasize the importance of reporting illumination conditions and gaze parameters in eye-tracking experiments to ensure data consistency and comparability. Our novel artificial eye provides a robust and reproducible platform for evaluating eye tracking systems and deepening our understanding of the human visual system.

## 1. Introduction

Eye tracking is a well-established, widely used technology across numerous scientific fields for investigating various biological and indirectly related psychological parameters regarding eye movements. Surprisingly, there is no consensus yet about the impact of the surrounding brightness conditions on the measurement of peak velocities in human saccades. Studies that addressed this issue reach differing conclusions about whether surrounding brightness conditions have an influence on saccadic peak velocities [1,2,3].

### 1.1. Motivation

There have been some statements claiming peak velocities in the dark would be slower than in the light [4,5]. This presents a challenge: often, they rely on old studies that did not even make said statement [6] or even reported the opposite when no peak velocity differences were found between saccades in the dark and a control condition without visual targets in the light [1]. Another issue arose from a comparison of memory-guided saccades (MGS) in the dark and visually guided saccades (VGS) in the light [7].

Since MGS have lower peak velocities than VGS [8], it is not advisable to compare them. In a previously employed investigation based on these discrepancies, we could confirm significantly lower peak velocities for MGS compared with VGS, regardless of the surrounding brightness conditions [2].

Furthermore, we discovered a non-linear change in peak velocities and durations for saccades made under three different brightness conditions, with the dark condition being the slowest, medium, the fastest, and bright in between them. Even after accounting for the variance explained by pupil size changes, the data still showed lower peak velocities for saccades in the dark and the light than in the medium brightness condition, thus remaining the same non-linear roof shape. The study was conducted using an EyeLink 1000 by SR Research, a popular video-based eye-tracking system [2].

**Figure 1:**
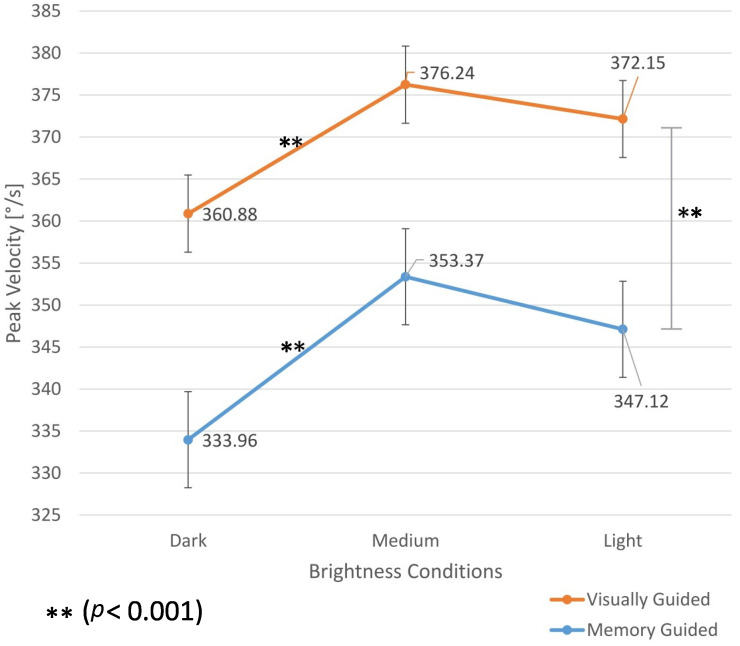
Mean peak velocities across participants for a 10° amplitude from our previous study [2].

The outcome of our initial study on the possible effect of the Brightness-Induced Roof Pattern (BIRP) on human saccades was challenged by peers [9], who questioned the source of these findings. This effect could be either human-related or an artifact of the eye tracker, potentially influenced by factors such as gaze position. The “roof-shaped” pattern of peak velocities can be observed in Figure 1.

To address these uncertainties and disentangle the potential confounds, we repeated the experiment using a novel approach. By developing and employing RELAY (Robotic EyeLink AnalYsis), a robotic artificial eye capable of executing precisely controlled saccadic movements under variable brightness conditions, we aim to achieve two primary objectives:

- Evaluate eye-tracking methodology: Test the accuracy and reliability of modern eye-tracking systems, such as the EyeLink 1000. This allows us to examine whether the system is capable of measuring reliable values or producing artifacts based on the technique itself.
- Examine BIRP effect: Investigate if our found differences in saccadic peak velocities are due to human factors, like pupil deformation or gaze position estimation, or some yet unknown cause. By removing the variability associated with human subjects, the RELAY system provides a robust platform for isolating and testing these effects.

Through this work, we aim to validate findings from our previous study, clarify the interplay between brightness conditions and saccadic dynamics, and ensure that future research is built on a solid methodological foundation. We expect the EyeLink 1000 to be unaffected by the brightness from the monitors, as the tracker relies on specific IR bands and uses filters to block other wavelengths, and the monitors should have a neglectable emission in the IR spectrum. However, the prior study reported brightness-related effects on saccadic peak velocity, raising questions about potential tracking artifacts. Since peers challenged these findings, our robotic eye experiment isolates human factors to verify whether the effect originates from the measurement system or the human visual system.

### 1.2. Background: Eye Tracking

Throughout history, there have been several methods to investigate the nature of human eyes and their movements, including electro-oculography (EOG), scleral search coils, photo-oculography (POG), and various video-based approaches such as video-oculography (VOG). Today, most modern eye trackers use video-based methods, with the dominant approach being pupil/corneal reflection tracking (P–CR), a subset of VOG. These systems track eye movements based on the pupil (P), the corneal reflection (CR), or, most commonly, a combination of both, as described below.

The video-based eye tracker identifies the pupil based on its contrast against the surrounding iris and tracks the CR as a bright spot caused by infrared (IR) illumination. The tracker emits IR light (typically via LEDs) directed at the subject’s eye, which interacts differently with the pupil and the cornea.

The pupil itself is an opening (aperture) in the iris and does not reflect light in the conventional sense. It appears dark because minimal light is scattered or reflected from within the eye. Its appearance depends on how IR light is illuminated and captured. In dark-pupil mode, off-axis IR light enters the eye and is mostly absorbed, making the pupil appear dark against the brighter CR. In bright-pupil mode, on-axis IR light is partially reflected from the retina, making the pupil appear bright.

**Figure 2:**
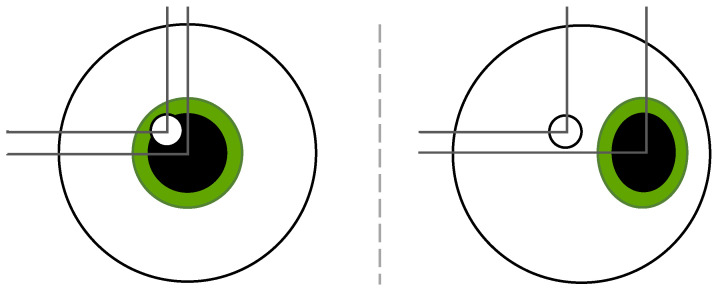
Lateral distance changes between corneal reflection (CR) and pupil based on gaze direction.

The cornea is the primary reflective surface, producing the first Purkinje image (P1), which is commonly used for gaze tracking. The eye tracker’s software processes grayscale signals to differentiate between the pupil and CR. The pupil’s grayscale intensity is determined in accordance with the natural light interactions and can vary from 0 (black) to 255 (white) depending on the mode selected by the tracking system. These two parameters, pupil position, and corneal reflection position, are analyzed together. When a subject looks straight at the eye tracker (typically positioned centrally below and in front of a screen), the P and CR signals are close to each other. As the subject looks away, the P signal moves while the CR remains relatively stable, resulting in an increasing distance between the two as the gaze shifts farther from the tracker. This principle enables precise gaze estimation. A visualization of this process is provided in Figure 2.

While the position of the CR is only slightly affected by the gaze position, the position of the pupil is highly dependent on it. If one wishes to track the pupil alone, head stabilization (e.g., a chin rest) is required to prevent movement artifacts. Otherwise, it would not be possible to distinguish between head and eye movements. In P–CR tracking, small head movements can be partially compensated by referencing the corneal reflection, though compensation is not perfect, and large head movements reduce tracking accuracy.

### 1.3. Background: Problems with Eye-Tracking

The manufacturer, SR Research, recommends using the EyeLink 1000 tracker in centroid mode for most applications. In this mode, the pupil’s position and size are calculated using a center-of-mass approach, assuming the pupil is approximately circular. This tracking algorithm is less vulnerable to noise compared with the ellipse-fitting approach, which is suggested to be used if the pupil is significantly occluded [10]. In centroid mode, the operator can choose to measure either the pupil’s area or diameter. Gagl, Hawelka, and Hutzler recommend using area measurements instead of diameter when analyzing both horizontal and vertical saccades. While the diameter option is more strongly affected by horizontal saccades, the area measurement is similarly affected in both directions [11].

One potential source of major impact on measuring is the pupil size. It is influenced by various factors, such as arousal [12], fatigue [13], stimulus intensity [14], cognitive workload [15], age [16], and, most importantly for this study, environmental illumination [17]. As stated by the last-mentioned source, decreasing brightness in the surroundings leads to an increase in pupil diameter, which can range from 1.5 mm to 8 mm.

While the before-mentioned influences on pupil size are relatively static, dynamic changes also occur during eye movements. For example, pupil deformation can happen during saccades due to the forces acting on the elastic material of the eye [3]. This deformation is often accompanied by a post-saccadic oscillation, also known as saccadic wobble. Such distortions can affect the estimation of the pupil’s size and shape, posing challenges for centroid mode, which assumes a circular pupil.

**Figure 3:**
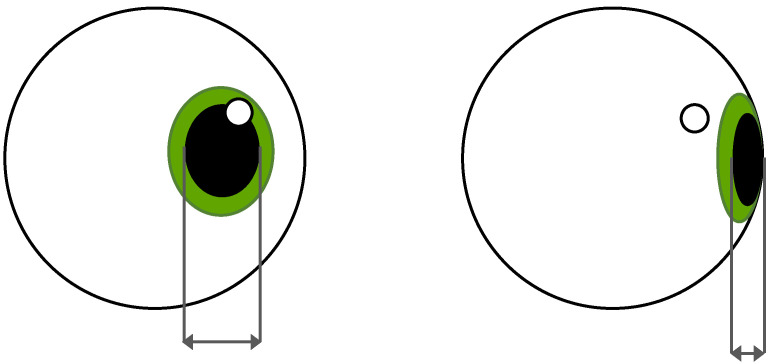
Pupil foreshortening effect: the observable shape of the pupil changes with gaze directions.

Furthermore, the gaze position alters the apparent shape of the recorded pupil from a circle to an ellipse, which can lead to difficulties in the estimation of its size and the exact gaze location [11,18]. An illustration of the change in shape is depicted in Figure 3. A possible contribution to this difficulty could be that the human pupil is not perfectly round, and thus, the estimation of its center shifts depending on the angle from which it is viewed.

**Figure 4:**
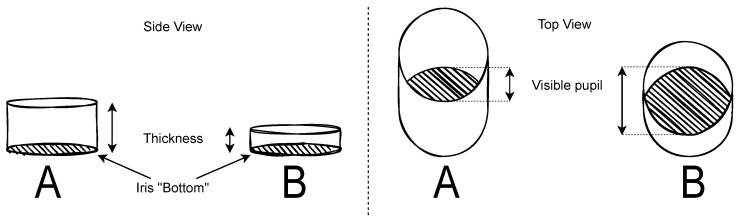
Different visible pupil sizes depending on the thickness of the iris (A: with thick iris, B: with thin iris).

Another impact on estimation difficulties of the size of the pupil and hence its position and velocity (as they are all correlated and the last depends on the others) is a fact that was already mentioned in 1962 [19]: “An additional source for a measurement error might be the anatomy of the iris (more specifically, the thickness of the iris) that defines the pupil”. A thicker iris does not cause any trouble while looking directly at the eye but leads to a stronger effect of apparent shape change in the pupil when looked diagonally at it, as it would have been if one looked at a tube. The seen ellipse shape becomes even narrower when looking at the hole (pupil) from the side when the iris is thicker than it would be for a slim iris [11]. Figure 4 depicts this effect.

The estimation of the pupil is relevant to drawing conclusions not only about gaze positions but also about peak velocities as well, as they are dependent on the first. It is important to consider these impact factors.

### 1.4. Relevant Works

One more obvious impact on the pupil size and, thus, probably the measured peak velocities that should be considered is the illumination of the surroundings. But despite the logical relation that they share, there is little research about the consequences of illumination on peak velocities. In 2016, Nyström et al. [3] reported similar results to ours [2] when they measured the peak velocities of four participants under seven different brightness conditions. Interestingly, they found the brightest condition (which led to the smallest pupil size) to produce the lowest peak velocities and the second-darkest condition the highest. The darkest condition again led to lower peak velocities. This was true for all measured signals that they used: CR, pupil, and gaze position, though to different extents. As a consequence, they also found a non-linear shape as a function of brightness. They interpreted the change in peak velocities in the gaze signals to be a function of the different pupil sizes. It is noteworthy that the changes in pupil size found in both studies were of a linear nature, but the changes in peak velocities were not. Therefore, pupil size cannot be the only explanation for the phenomenon.

In a recent work, researchers investigated a phenomenon called the Pupil Size Artifact (PSA), which is an apparent gaze deviation that occurs when the pupil changes its size, although the eyeball does not rotate (for instance, during fixations) [20]. This was also denoted by Wyatt [21], who stated that when pupil size changes, the center of the pupil can shift, which affects the gaze position estimation, as the eye tracker seeks to determine the position based on it. These different studies show that the results and, thus, the agreement on the metrics of saccadic peak velocities in dependency on surrounding illumination and pupil sizes are not given to this day. There is no consensus regarding the influence of brightness conditions. It becomes evident that it is a very delicate topic to try to disentangle the multiple influence factors on something that is, in theory, such an easy principle.

While it is true that the pupil size indeed can be a factor, we will ask here how it is the other way around. To what extent do the surrounding brightness and the gaze position have an influence on the pupil size of humans and therefore the measured peak velocities? To deal with this question, we aimed to eliminate the influence caused by the pupil size by utilizing an artificial eye with a constant pupil size in a robotic motion system.

Furthermore, we controlled the velocity of the robotic eye’s movements, the gaze positions, and the surrounding illumination. As a result, we can draw conclusions about the impact of the gaze position and the brightness conditions on human saccades.

If our previous findings regarding peak velocity changes with different brightness conditions are based on a human factor, our results in this study should show no different peak velocities for different brightness conditions when the robotic eye performs saccades of a certain amplitude with predefined velocity. We used the EyeLink 1000 by SR Research Ltd. (Mississauga, ON, Canada), as it is a commonly used, state-of-the-art system.

Our constructed system, which will be described in the subsequent section, is the **R**obotic **E**ye**L**ink **A**nal**Y**sis apparatus, henceforth referred to as RELAY.

## 2. Methods

### 2.1. RELAY Robotic EyeLink AnalYsis

The idea is to use a mobile, lightweight, precise, and programmable apparatus with an artificial eye to mimic representative, human-like saccades. To keep the artificial eye fixed in place while allowing predefined movements along two axes, a specialized hinge is required.

**Figure 5:**
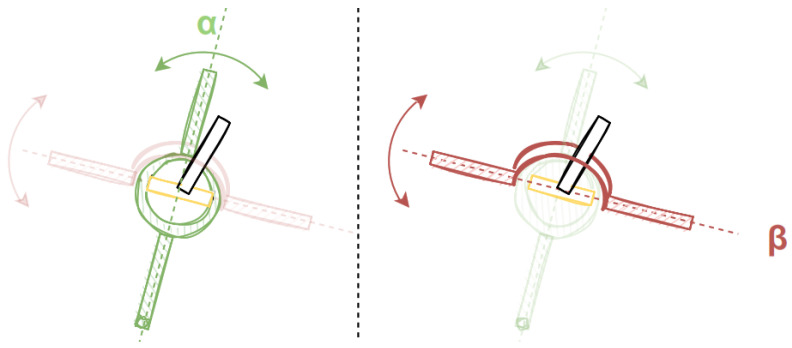
Double pivot hinge that allows unrestricted movement in two axes, α and β.

The mechanical challenge behind this problem is that the rotation axes need to have a common intersection point in order to achieve a superposition that is free of mutual influence. A solution for the challenge of the intersecting axis can be seen in Figure 5.

The first axis α, depicted in green, is moved by a pivot lever, depicted in black, which is connected to the axis β by a perpendicular hinge (yellow). Due to the orthogonality of the hinge, the rotation parallel to α gets transferred directly to the axis, while other rotational movements get canceled out by the hinge.

The superposition of two orthogonal axes in the dual-axis hinge moves the black lever to a desired position. Using this idea, the mechanical build is based on two stepper motors, one for the X-axis and one for the Y-axis of the eye, connected to an analog stick (working as the dual-axis hinge) from a gamepad, that allows an axis unrestricted movement. The analog stick also contains built-in potentiometers for each axis, which is necessary for initial calibration.

The two used NEMA 17 (42 mm × 42 mm × 47 mm) stepper motors (QSH-4218-35-10-027 and QSH-4218-41-10-035 from TRINAMIC Motion Control GmbH & Co. KG, Hamburg, Germany), typically found in 3D printing or computer numerical control (CNC) applications, were paired with an Arduino board and a stepper driver expansion shield and driven in 16-micro-stepping mode, resulting in a smooth and quiet motion and a theoretical addressable resolution of 0.1125° per micro-step.

The positioning error, considering the potentiometers, Arduino analog-digital conversion, and the used stepper motors and drivers, can be estimated as ±2 micro-steps or ±0.225°, not including backlash or mechanical imperfections.

The choice for the artificial eye fell on a synthetic eye prop made for professional theater acting purposes (GRIMAS B.V., Heemstede, Netherlands) [22]. It has a total size of 27 mm with a 15 mm iris and a 5 mm pupil. The artificial eye features a layer that is comparable to a real cornea but, of course, rigid. Another point that led to the used eye was the fact that it was easy to obtain and mount to the mechanical built.

**Figure 6:**
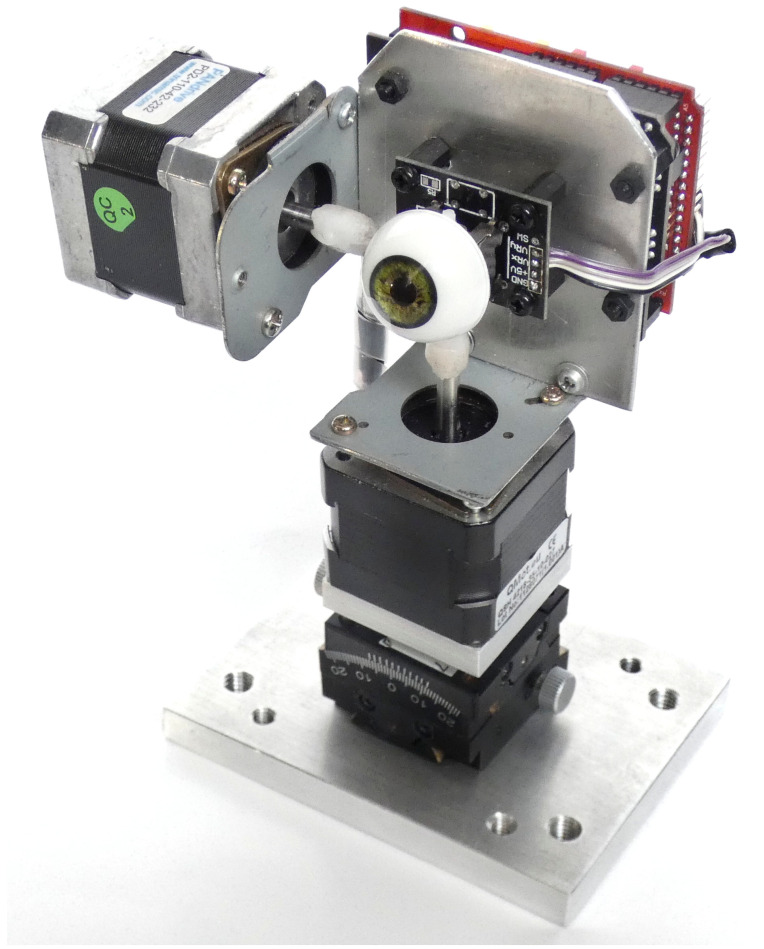
The final RELAY assembly: two stepper motors connected to an analog stick with an artificial prop eye. An Arduino UNO with a CNC expansion shield and two stepper drivers powers the motors (12v). The lower motor is attached to the base using a dual tilt adjustment adapter.

The resulting apparatus has the eye-to-base plate distance of 11 cm, which is a typical human eye-to-chin distance, allowing RELAY to be used with an unmodified chin rest of an eye tracker. Detailed assembly information, a parts list, and Arduino code are published as an open-source git repository [23]. The final RELAY assembly can be seen in Figure 6.

### 2.2. Experimental Setup

The experiment was run in the eye-tracking laboratory of the Otto-von-Guericke University in Magdeburg, Germany. The build-up with the eye tracker and the participant monitor was enclosed in a soundproof cabin, thus leaving the surrounding very well controlled on behalf of the illumination conditions because it was possible to set the display illumination as the only light source.

**Figure 7:**
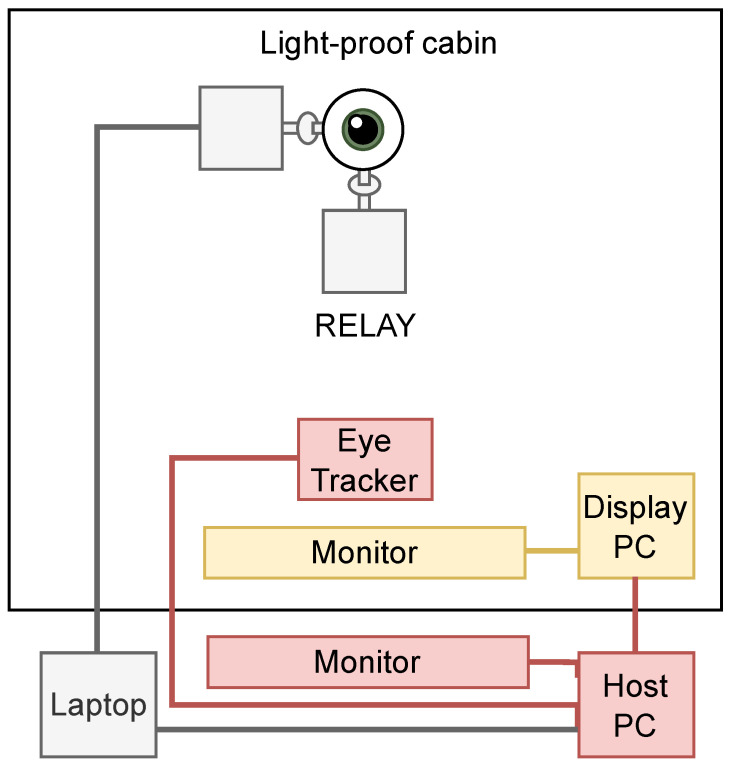
Schematic overview of the test setup.

The host PC (that runs the eye tracker) and a laptop from where the experiment was controlled were placed in the laboratory outside the cabin. A schematic overview containing the connections between the different components can be seen in Figure 7.

The laptop (2.60 GHz, 16 GB RAM, 1 TB SSD, Windows 10) on which the experiment code was run, using the Psychophysics toolbox version 3 [24] for MATLAB^®^ (Mathworks, Natick, MA, v. R2020b), was connected to the host PC and the display PC. It was further connected with the Arduino control unit, on which a program written in C++ was run to control the movements, and a PlayStation^®^ 4 controller (Sony Group Corporation, Minato, Tokyo, Japan) to achieve better ergonomics for starting different RELAY modes corresponding to a button press. The scripts and program code used in the experiment can be found here [23,25].

Two ProLite GB2488HSU-B1 (iiyama, Nagano, Japan) 24-inch monitors (298.89 mm × 531.36 mm, 1920 × 1080 pixels, 144 Hz) served as displays for host and participant PCs. The distance from the top knob of the eye tracker to the chin rest was 530 mm, which is in line with the recommended minimum distance when using an 890 nm illuminator [10]. The bottom knob of the eye tracker was centered horizontally along the monitor. The measurement technique used was PC-R, as it is not susceptible to head—or, in this case, robotic—movement errors as the other techniques. In accordance with the statements of Gagle et al. [11], we used the eye tracker in “centroid mode” with the pupil area option to ensure that the signal noise was low for the used gaze directions, making the results comparable to other studies that mainly use these options. The sampling rate was 1000 Hz, and standard saccade detection criteria from SR Research were applied.

**Figure 8:**
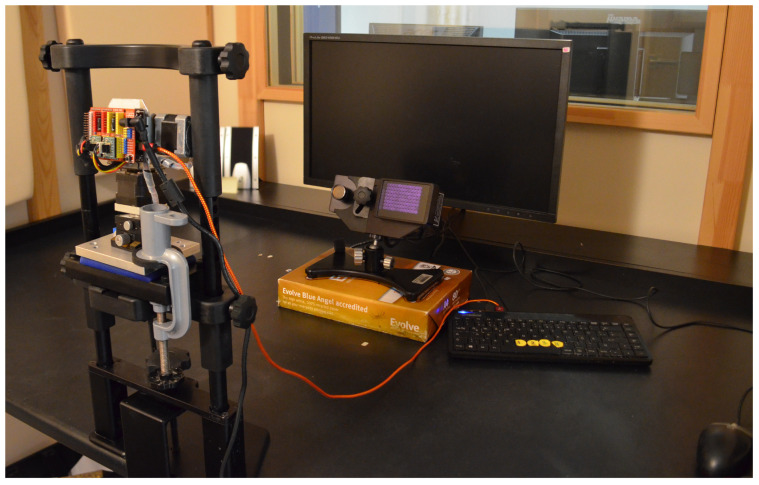
Setup in the soundproof cabin. The operator sat on the other side of the glass window, which was occluded for the experiment.

The RELAY system was clamped to the chin rest and positioned in such a way that the artificial eye would approximately be in the same position compared with a human participant, 29 cm above the table. The chin rest height slider and the RELAY tilting adapters were then used to bring the apparatus to a realistic position, right in front of the monitor. The view of the cabin and the experimental setup can be seen in Figure 8.

The distance from the artificial eye to the screen was set to 927 mm, according to the recommended distance of at least 1.75× the width of the display area by the manufacturer [10]. This was conducted to fulfill the convention, as it was strictly speaking not necessary for this experiment, as the artificial eye is “blind” and thus does not see the monitor. However, it was important to obtain the right screen coordinates in pixels for the 13-point calibration grid that was applied before testing and to be able to obtain saccades of the desired amplitudes. The required coordinates for the calibration grid were calculated with the SR research calibration coordinate calculator [26].

### 2.3. Accuracy and Precision

A key element for the success of the experiment is that RELAY has to move precisely and accurately. Precision, in a narrower sense, means that on multiple measurements with identical settings, the results should also be close to each other (ideally identical). The more alike the results are, the better. Accuracy is given when a measurement leads to results that are very close (ideally exactly) to the true value (τ). For our moving artificial eye, this means that when RELAY is moving frequently towards a predefined, planned position, it should always move in the same amount, resulting in close values for the position, to ensure precision.

Furthermore, when the measured position matches the desired position, then it is accurate. If the movements are not precise, there is no way to control the recorded values by the eye tracker at all. The input (predefined movements) must be reliable to attain a reliable output (measured movements). If the precision is known and sufficient, every other parameter in the system can be adapted in order to become accurate as well. This is the base on which the whole system can be calibrated and validated by the eye tracker; thus, it had to be tested first.

To ensure adequate comparability with human saccades and still have good controllable, smooth kinematics, the speed of RELAY’s movements had to be determined. The motor speed, acceleration, and deceleration are controlled by the driver by increasing or decreasing the pulses per second (pps), which correspond to a micro-step until the desired speed and position are reached. Trials with speeds of 100, 1000, 3200, and 5000 pps were run. For the acceleration, a value of 80,000 pulses per second² was set. With 100 and 1000 pps being too slow for the eye tracker to correctly detect saccades (they were interpreted as microsaccades) and 5000 being too fast, as it resulted in problematic overshooting out of the monitor screen and thus the trackable range, 3200 pps were chosen. Converted into degrees per second, which results in 360°/s.

Seven patterns were chosen for the accuracy and precision experiment: a horizontal and a vertical line (consisting of several saccades of differing length along the X- and Y-axes, respectively), multiple horizontal lines along the screen, multiple vertical lines along the screen, a diagonal cross (“X”), and a 13-point calibration grid. The 13-point grid was chosen, as advised by the manufacturer, due to the fact that we were using a rather large calibration region to calibrate the eye tracker. The last one was a complex pattern consisting of 160 random saccades.

### 2.4. Laser Experiment

**Figure 9:**
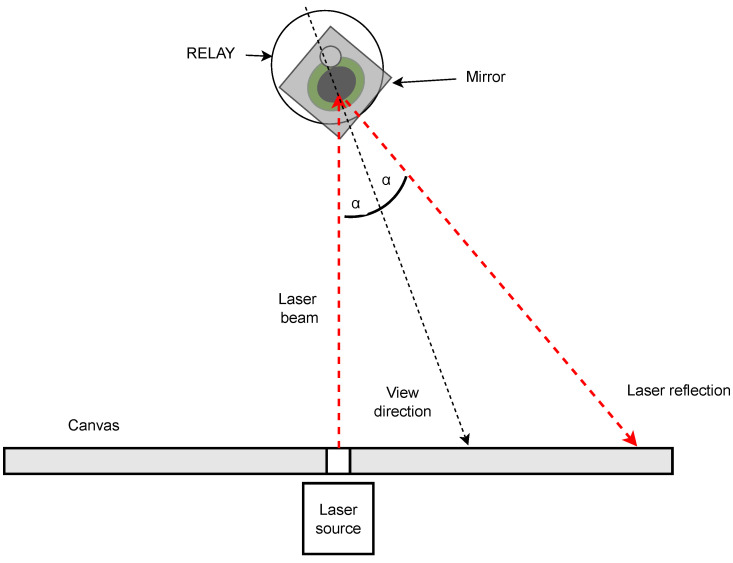
Laser beam reflected by the mirror on the eye.

After recording the different patterns with the eye tracker, a second run was conducted using a laser dot and a mirror. For this, a canvas with a laser diode in its center was placed in front of the eye. The laser was aimed directly at the middle of the artificial eye. The eye was equipped with a little front surface mirror that reflected the laser back onto the canvas depending on the gaze position of the eye. It was adjusted so it would reflect the laser back to the source in the neutral position. A schematic depiction of the principal function of the laser measurement setup is shown in Figure 9.

The patterns drawn by the laser on the canvas represent the viewing direction of RELAY in an enhanced manner, as the angle of the laser reflection is double the angle of the viewing direction of the eye. This is caused by the law of reflection for this case of specular reflection, where the reflected ray of light (the laser pattern on the canvas) emerges from the reflecting surface at the same angle to the surface normal as the incident ray, namely the laser input. The surface, normal, in this case, is the mirror attached perpendicular to the viewing direction.

**Figure 10:**
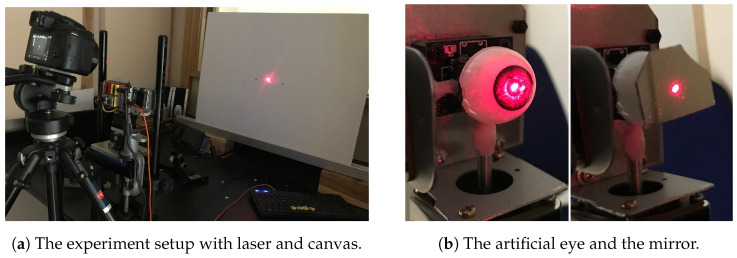
Laser experiment setup.

The resulting patterns on the canvas capture the movements of the artificial eye with great detail due to the trigonometric relations between the canvas, laser, and mirror. With a Lumix DC-FZ83 camera (Panasonic Corporation, Osaka, Japan), pictures were taken in long-time exposure mode, making the patterns visible. With this procedure, it was possible to compare the planned patterns to the actual patterns that were performed by the robot and the patterns that the eye tracker recorded. The pictures from this procedure are referred to as laser pictures and can be seen in Section 3. For the precision measurement, RELAY performed 100 trials of the single horizontal line, the single vertical line, and the calibration grid in order to compare the raw data sets and the calculated saccades for each pattern. Figure 10a,b shows the laser measurement setup.

### 2.5. Artificial Saccades Experiment

**Figure 11:**
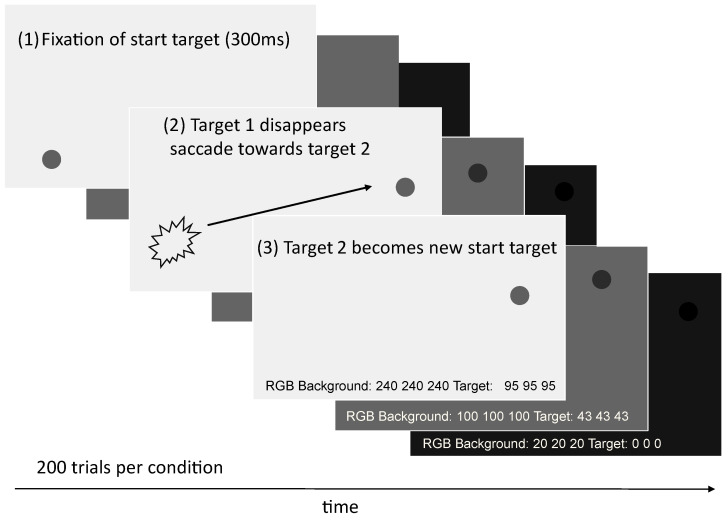
Visualization of the trial design for all three brightness conditions.

The experimental run followed the design of our initial study [2], but only for the VGS. This means that the saccades by RELAY were performed with the same three RGB (red, green, and blue) value settings (see Figure 11) for the background and the targets on the screen as before, leading to similar brightness conditions dark, medium, light).The conditions are summarized in Table 1.

As the new experiment was conducted with another monitor, the luminance and contrast measurement had to be repeated. The luminance of the display was measured with a Chroma Meter CS-200 (Konica Minolta Sensing Europe B.V., München, Germany) on five positions of the display (all corners and the center). From these values, the mean was calculated.

Together with the measured luminance values for the target dots, Weber contrasts were calculated. The resulting measured and calculated contrast values were 0.68 (dark), 0.85 (medium), and 0.83 (bright), which is slightly different from our initial experiment with human participants, where a similar contrast for the three significantly different lighting conditions was emphasized [2]. Nonetheless, it was decided to use the same RGB values in order to make them comparable. For our experiment with RELAY, contrast is not expected to influence the results, as the artificial eye is blind and follows pre-programmed positions rather than visual stimuli. The key factor is the presence of three distinctly different brightness conditions, which were successfully achieved. The experiment was conducted with an EyeLink 1000, with the same settings as described in Section 2.2.

The experiment was designed to mimic 200 participants doing 200 saccades per condition, resulting in a total of 120,000 saccades (40,000 per condition). As in our initial experiment with human participants, after the artificial eye fixated on a target dot for 300 ms, the dot disappeared, and the next target appeared. This was followed by a saccade to the second dot (with 3200 pps), which became the new starting point for the upcoming saccade after another fixation for 300 ms and so on. The 3200 pulses per second correspond to 360°/s, as explained in Section 2.3. After initial calibration and validation to the 13-point calibration grid, the experiment was run for several hours without any interruptions so that the calibration only had to be conducted once.

The positions of the dots were calculated randomly for every *participant*, leading to saccades in all kinds of directions, ranging from 4.5° to 14.1° of visual angle. The distances of the target points were chosen to correspond to the possible range minus 10% so that if overshoots occurred, the saccades would not protrude from the screen. Over the three conditions, the positions stayed the same for every participant in order to make them comparable to each other. Figure 11 explains the sequence which was also used in our prior experiment with real human subjects.

## 3. Results

### 3.1. Accuracy: Laser Patterns

**Figure 12:**
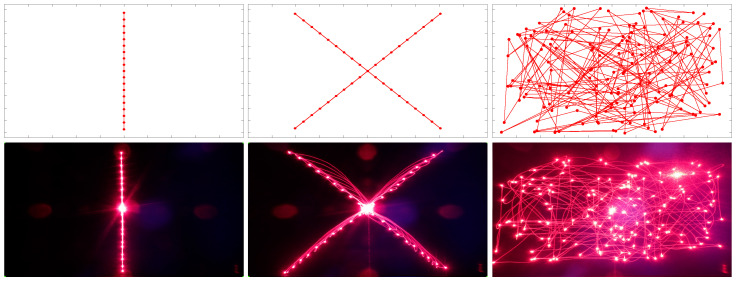
Planned patterns vs. long exposure laser patterns.

The distance from RELAY to the canvas was 70 cm when the long-time exposure pictures were taken. For the simple patterns, the distances between every position (seen as red dots in Figure 12) were set to eight micro-steps, giving 0.9° each. For example, in the case of the single horizontal line, it summed up to 15 steps to the left and 15 steps to the right. This corresponds to theoretically 27° (or 33.6 cm) in both directions for the outermost coordinate (note that the angle is doubled because of the mirror on the artificial eye, as explained in Section 2).

With the dimensions of the canvas of 67.9 cm × 49.8 cm, it is possible to calculate the real driven angle by the robot. The rightmost point is directly at the edge of the canvas, i.e., exactly 33.95 cm from the center where the laser input is. Hence, the deviation of the theoretical and the real coordinates is 0.35 cm, respectively, and 0.29° of visual angle.

When drawing conclusions about the accuracy based on the taken pictures, it is important to notice the different angles of the camera and the eye towards the canvas (parallax), as it could only be put somewhere close to the eye but not on the exact same spot, lead to a small distortion of the taken pictures. Another distortion, a so-called barrel distortion, originated from the objective lens of the camera. To compensate, image registration with perspective correction was conducted using GIMP 2.10.22 [27].

In Figure 12, a comparison of the planned patterns and the corresponding laser pictures can be seen. The planned patterns show the simulated resulting reflection based on the trigonometric values for the distance, canvas size, and the original functions, as used to program RELAY.

The accuracy of the planned 13-point calibration pattern versus its raw data from the eye tracker was evaluated by calculating the absolute deviation of the measured X- and Y-positions’ mean during fixation periods over 100 trials ($\bar{x}$) from the planned true coordinates (τ). This allowed the determination of the absolute deviations in degrees of visual angle, reflecting the accuracy. Table 2 shows the results. Deviations for the calibration grid ranged from −13.3px to +38.5px (or −0.23° to +0.66°) for the X-axis and from −18.5 px to +17.4 px (or −0.32° to 0.30°) for the Y-axis.

**Figure 13:**
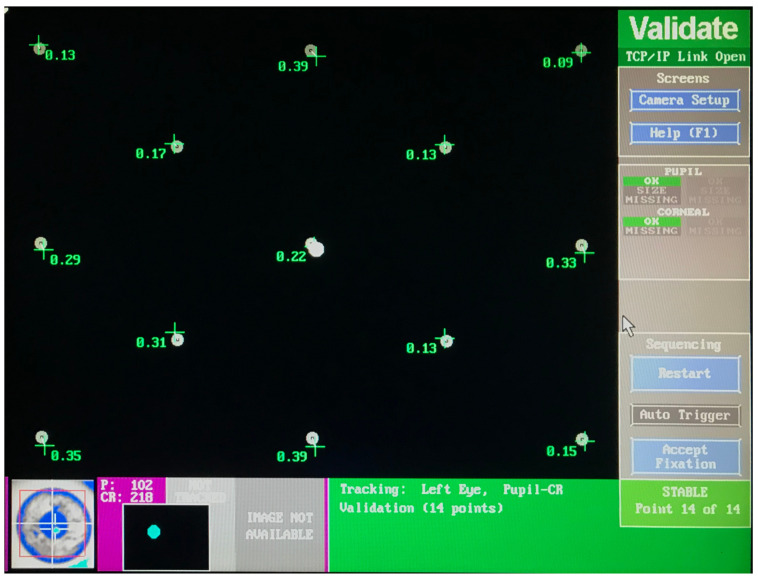
Validation of the 13-point calibration pattern as used by the EyeLink 1000 Eye Tracker.

This pattern and the positions were also used when calibrating RELAY with the EyeLink 1000 tracker, using the settings described in the artificial saccades experiment in Section 3.4. Figure 13 shows the measured gaze position variation ranging between 0.09° and 0.39°, which is better than a typical measurement for humans at around 0.5°.

**Figure 14:**
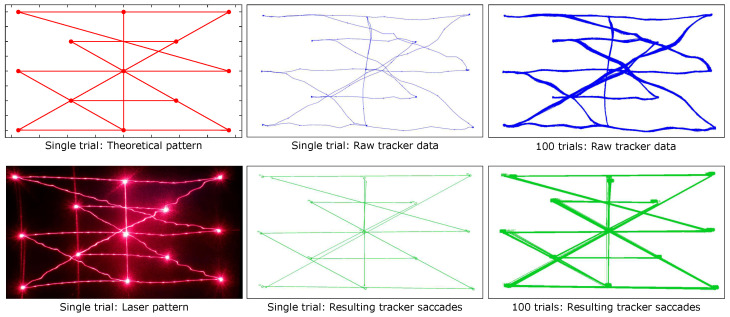
Eye tracker data for 13-point calibration pattern, including theoretical, laser, raw, and calculated saccade values.

The corresponding laser picture with the planned and the real pattern can also be seen in the left panels of Figure 14 in the upcoming Section 3.2.

### 3.2. Precision

The first step of precision analysis was performed with the usage of the software Data Viewer, Version 4.1.211, by SR Research. This program is able to show the trial data either in a 2D temporal graph mode or in a spatial overlay view, based on the recorded coordinates. It makes it possible to see all recorded gaze positions and the movements between them of a single trial at once (Note: in this case, one calibration pattern is viewed as one trial).

The spatial overlay view of the program can be used with the recorded raw sample data or the performed saccades, which are based on a calculation from the first performed by the program. Furthermore, the positions and durations of fixations are observable. This can come in handy to provide a good overview of the data. The SR Research Data Viewer was used to overlay calculated saccades from 100 precision trials for the 13-point calibration grid. Sadly, there was no option to do the same with the raw sample data sets, so they had to be extracted for a manual overlay. This was again carried out with the use of GIMP [27].

Figure 14 compares the planned and real laser 13-point calibration grid pattern (left part), the raw data and the calculated saccades for its single trials (middle part), and the overlays of 100 trials for the raw and calculated saccades (right part). The accumulated overlays for the horizontal and vertical lines look similar in quality and can be seen in the Supplemental Materials [25]. One can intuitively draw conclusions on the precision from the similarity of the accumulated data, as the shape does not differ much from those of the single trials.

We calculated the spatial precision of the eye tracker and RELAY for the 13-point calibration pattern, using the standard deviations to the means from the raw data’s X- and Y-positions over all 100 trials (during fixation periods). We also converted the standard deviation values for the X and Y positions into millimeters and degrees of visual angle, respectively, which provided a metric for the precision of the combined eye tracker and robot system. The standard deviation values for the precision ranged from 0.3 px to 2.2 px (or 0.005° to 0.04°) for the X-axis and from 0.7 px to 2.6 px (or 0.01° to 0.04°) for the Y-axis. Table 3 shows the values for all 13 coordinates.

### 3.3. Comparing Saccadic Profiles

**Figure 15:**
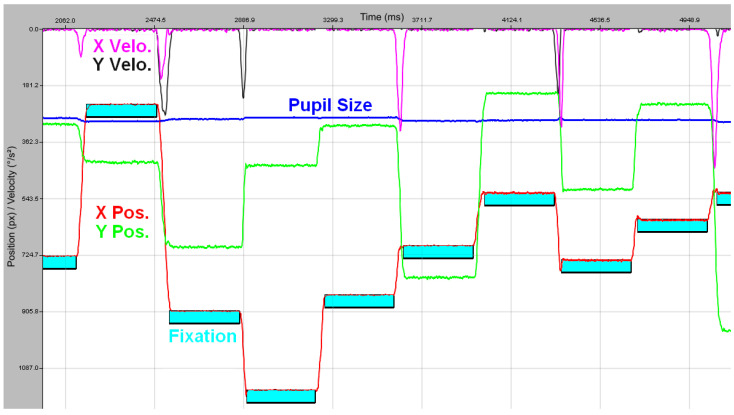
Temporal Graph View (EyeLink Data Viewer 4.4.1) of a random trial from the Artificial Saccades Experiment. The X-axis represents time, while the Y-axis displays multiple gaze-related parameters: X (red) and Y (green) gaze locations in screen pixels, pupil size (blue, arbitrary units), and velocity data in degrees per second for the X-axis (violet) and Y-axis (dark gray).

To validate RELAY’s artificial saccades, we compared its velocity profiles to known human saccade dynamics. Figure 15 shows an excerpt from the experiment with random oblique saccades as a temporal graph view with raw data.

**Figure 16:**
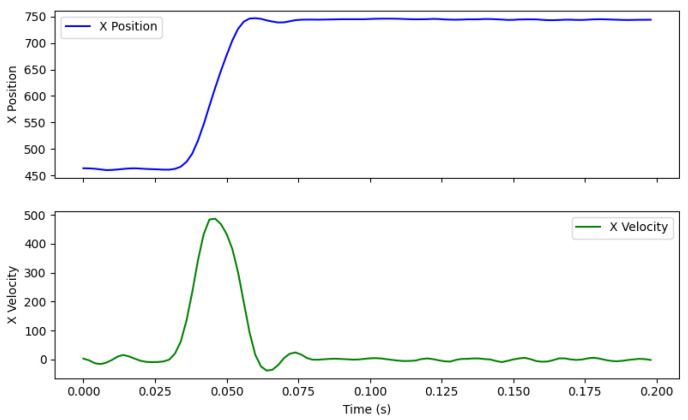
Saccade waveforms for position and velocity signals.

After implementing the same data analysis approach as Nyström et al. (2016, Section 2.4) [3], we detected saccades using velocity outliers, defined as at least six consecutive samples exceeding a velocity threshold $\eta_{x,y}=\lambda \sigma_{x,y}$, with λ=3 and $\sigma_{x,y}$ representing the estimated noise in the velocity signal for the horizontal and vertical directions [28]. Velocity was computed as the first derivative of position using a 3rd-order Savitzk–Golay filter with a seven-sample window. This analysis produces a saccadic profile comparable to that of humans, which is illustrated in Figure 16. The resulting saccadic velocity follows the characteristic, symmetric bell-shaped curve observed in human saccades, as described by Bahill et al. [6]. These findings support RELAY’s use for evaluating eye trackers without introducing non-human motion artifacts.

### 3.4. Artificial Saccades Experiment

A total of 120,000 saccades should have been recorded (200 trials for each of the 200 artificial participants), but two “participants” had to be excluded from the analysis because the data set, however, was empty. The saccades were aimed to lie between 4.5° and 14.1°, where the human main sequence has a linear shape [6]. If one adds the acknowledged tolerance of 0.5° [10], the range becomes 4° to 14.6°. Saccades recorded during transitions between “participants” were always excluded, as their amplitude sometimes exceeded the intended size due to random start positions. Another noticeable issue was that the tracker occasionally lost track of the eye (possibly due to a reflection or the end of the trackable range) during saccades that had approached the upper right corner. This caused an abort of the measured saccade, which was split into two smaller ones. Those were also excluded. The described exclusion criteria resulted in a remaining 115,681 saccades (96.9%).

First, we plotted a main sequence for all “participants” across all trials and conditions to verify whether it resembled human-generated saccades and to obtain an initial overview. Using linear regression with saccade amplitude as a continuous independent variable, we estimated a peak velocity of 357.9°/s for a 10° saccade, an amplitude chosen because it falls within the linear range of the main sequence, ensuring comparability across conditions and participants. The resulting plot closely resembled a typical human main sequence [6]. The same analysis approach with 10° saccades was also used in the previous study. However, due to the large number of data points and overlapping values, the plot became visually overloaded and did not provide additional insights, so we included it in the appendix [25].

Next, we generated separate main sequence plots for each condition overall participants (dark, medium, and light) and performed linear regression for each, treating amplitude as a continuous variable. This allowed us to estimate peak velocity as a function of amplitude without restricting it to predefined amplitude categories. Specifically, we extracted the estimated peak velocity for a 10° amplitude to compare conditions. The results were nearly identical across conditions: 357.908°/s (dark), 357.911°/s (medium), and 357.900°/s (light). These plots for all participants and conditions with the regression lines are also included in the appendix [25] for the same reason as above.

**Figure 17:**
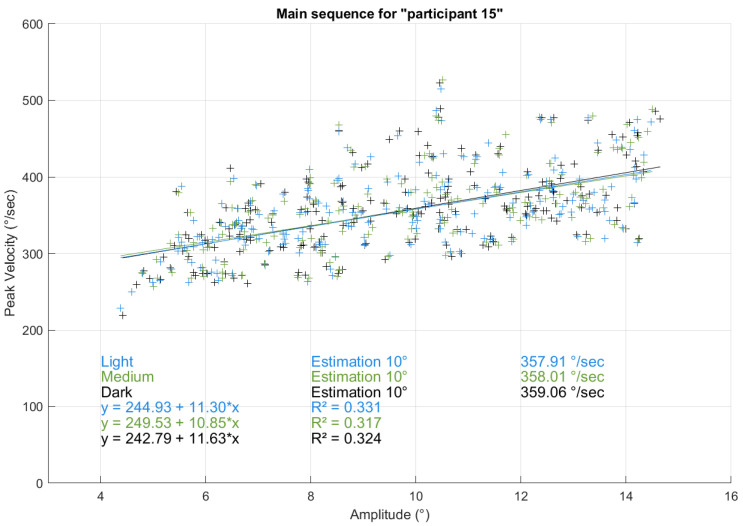
Main sequence for random “participant 15” overall conditions. All three linear regressions lie close to each other.

For illustration purposes, as an example, Figure 17 depicts the main sequences for a randomly chosen “participant” for all three brightness conditions. It becomes evident that the data points over the three conditions lie close to each other, as they should because every condition used the same coordinates and thus led to similar variances of amplitude and peak velocities. The main sequences for all other “participants” with the corresponding regression values were plotted as well and looked similar. They can be found in an animated Graphics Interchange Format (GIF) in the appendix [25].

The plots indicate again that the brightness conditions seem to have no influence on the measured peak velocities. The estimated peak velocity values for an amplitude of 10° for this “participant” are 357.91°/s (dark), 358.01°/s (medium), and 359.06°/s (light) and are close to the overall values, as for all “participants”. The R² metric for this given “participant” is 0.324 overall (0.324, 0.317, and 0.331 for dark, medium, and bright conditions), indicating a very similar amount of explained variance over the conditions. Among “participants”, we found the R² metric ranging from 0.2 to 0.491, but always very similar between the conditions. Since the deviance is only among the “participants” and not the conditions, the source must be of a systematic nature, e.g., the gaze positions or motor imperfections. It should be taken into consideration that this metric was calculated for all types of saccades that were made (horizontal, vertical, oblique).

To test whether the brightness conditions had an influence or not, the estimated peak velocity values for the amplitude of 10° were entered in a repeated measurements analysis of variance (rm ANOVA), with the factor brightness and the three levels dark, medium, and light. The rm ANOVA confirmed that there was indeed no difference between the brightness conditions, which is indicated by the within-subjects (or between treatments) effect failing to reach significance: *F*(2; 394) = 0.015, *p* = 0.985, partial $\eta^{2}$ < 0.001.

Additionally, it was tested whether the recorded pupil sizes differed significantly in dependence on the brightness condition. For further evaluation, the measured values were also entered in a rm ANOVA with the same factor and levels. A Mauchly test showed a violation of sphericity; therefore, the Huynh–Feldt adjustment was used to correct it. With *F*(1.993; 74,423) = 0.115, *p* = 0.891, and partial $\eta^{2}$ < 0.001, the within-subjects effect again failed to reach significance, which shows that the pupil sizes did not differ for the brightness conditions.

The initial hypothesis can be stated as follows: If there is a brightness-related artifact caused by the eye tracker, it should result in different measured peak velocities and pupil sizes across the three different brightness conditions given the same predefined amplitudes and velocities. Since all brightness conditions led to the same measured peak velocities and pupil sizes, it can be assumed that the brightness of the environment has no influence on the peak velocity nor on the pupil size measurement of our robotic eye setup.

### 3.5. Pupil Size vs. Gaze Direction

**Figure 18:**
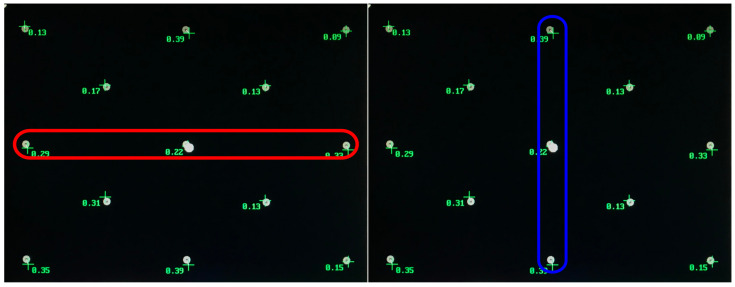
Trials from 13-point calibration grid split into two groups evenly. Half trials for each horizontal rm ANOVA (red) with three levels (left, middle, and right) and vertical rm ANOVA (blue) with levels (up, middle and down).

To examine whether gaze direction affects the measured pupil size, we analyzed the pupil size data recorded at the outer medial and the center coordinate of the 13-point calibration pattern. This approach was eligible because the coordinates were precisely known, allowing us to categorize the pupil data into gaze direction groups. The resulting directional groups were divided into horizontal (with three levels: left, middle, and right) and vertical (with three levels: up, middle, and down). Figure 18 shows the points used for groups horizontal and vertical with their levels.

Each of the three coordinates provided 100 pupil size measurements, resulting in a total of 300 values per direction group. The pupil size values (given in the tracker’s arbitrary units) were assigned into their respective directional groups based on their position on the screen. We used values for horizontal analysis where the vertical axis is constant and vice versa, namely leftmost middle, middle, and rightmost middle for horizontal and upmost middle, middle, and downmost middle for vertical. This ensured that there was no confound in regard to the gaze directions.

**Figure 19:**
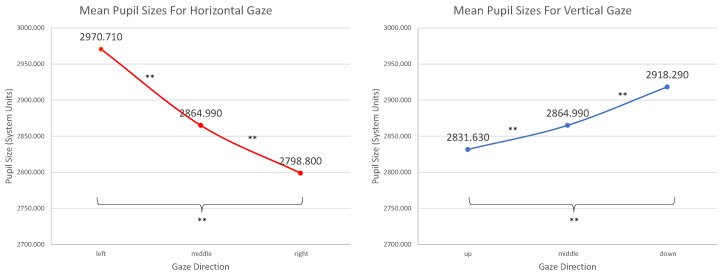
Mean pupil sizes from the rm ANOVAs for the gaze direction groups. Error bars indicate standard error. Double asterisks indicate highly significant mean differences.

The pupil size values were entered into two separate rm ANOVAs with one factor (horizontal or vertical group) and three levels for each direction. In Figure 19, the mean pupil sizes as a function of the gaze directions can be seen.

We conducted a Mauchly test for sphericity to assess whether measured pupil sizes varied systematically with gaze direction. For horizontal gaze directions, the test revealed a violation of sphericity, necessitating a Greenhouse–Geisser adjustment, *F*(1.788; 177) = 104,108; *p* < 0.001, partial $\eta^{2}$ = 0.999. Post-hoc, pairwise Bonferroni-adjusted comparisons confirmed significant differences in pupil size between all horizontal gaze groups (left vs. middle, middle vs. right, and left vs. right; all had *p* < 0.001). Pupil size was largest when gazing left and smallest when gazing right, with intermediate values for central gaze. Similarly, the Mauchly test indicated a violation of sphericity for vertical gaze directions, requiring another Greenhouse–Geisser adjustment, *F*(1.873; 185) = 43,709; *p* < 0.001, partial $\eta^{2}$ = 0.998. Post-hoc, Bonferroni-adjusted comparisons revealed that pupil size was significantly larger when gazing downward compared with middle or upward directions (*p* < 0.001). Another post-hoc, Bonferroni-adjusted comparison revealed that pupil sizes differed significantly between all vertical gaze directions (up vs. middle, middle vs. down, and up vs. down; all had *p* < 0.001).

These results suggest that there is a bias for measured pupil sizes depending on the driven coordinates and gaze positions.

## 4. Conclusions and Discussion

This work had two purposes: The first was to evaluate the accuracy and precision of RELAY and the EyeLink 1000. The results from the laser and calibration patterns (see Figure 14) show an overall superb precision and accuracy for the RELAY apparatus as well as the EyeLink 1000 tracker. The theoretical patterns were correctly represented in both the long-term laser pattern exposures and the resulting saccades, calculated by the eye tracker. These results indicate high suitability for RELAY as a device for testing the EyeLink 1000 in particular and methods for video-based eye tracking in general.

The RELAY apparatus can be compared with artificial eye platforms used by Reingold [29] or Wyder et al. [30] for their respective eye tracker accuracy measurements. Reingold [29] used a modified pan-tilt platform designed by Schneider et al. [31], which was initially used as a camera gimbal for their “Eye See Cam” experiment. Reingold states the accuracy of the platform as approximately 0.85°, which is not enough to do an automatic calibration with the tracker, that is why a manual calibration with the help of a laser attached to the platform was used. The laser beam was used to align the platform to defined points and thus resulted in a “gaze position accuracy [variation] between 0.02 to 0.05 of a degree” [29]. RELAY was able to move to the desired calibration positions with sufficient accuracy automatically, without any manual help. Unfortunately, the platform used Schneider et al. is not open source or depicted in detail for reproduction.

Wyder et al. used a four degrees of freedom (4 DOF) apparatus with an artificial glass eye capable of pan/tilt and linear X/Y motion. The eye holder was precisely manufactured with the help of a CT scan of the eye and has a checkerboard for visual calibration. The precision goniometer, linear micro-stages, worm-geared rotational stage, and a calculated calibration model for mechanical imperfections allow accurate positioning of the eye, which they then consider ground truth for the eye tracker. This apparatus, however, is only capable of static measurements; as the tilt mechanic, the goniometer has to be operated manually, and the worm-geared rotation stage is too slow to simulate saccadic eye movement [30].

When compared with the currently available solutions for mechanical eye tracker evaluation, RELAY offers a possibility for not only static positioning but also for saccadic eye movement comparable with those of humans. RELAY can be easily reproduced with parts commonly used in DIY 3D printers. The source code and further in-depth explanation can be found on GitHub [23]. RELAY is meant as an initial starting point and an affordable platform that can easily be improved by using better stepper motors and drivers. It is also possible to improve the code to compensate for hardware imperfections. The dual-axis hinge from the game controller analog stick, while working reasonably well, is not meant to be used “in reverse”, i.e., using the motors to drive the potentiometers and thus moving the pivot element. This mechanical part can be identified as the main source for (non-linear) backlash and thus could be improved in further approaches. The apparatus is the size of a human face, so there would be no problem using it for different kinds of eye trackers, including head-mounted, after proper mechanical adaption. We encountered the limitation of only having one eye tracker for testing available, but with some adaptation, it should be possible to track other video-based eye tracker systems as well.

Recent studies have employed RELAY as a controlled apparatus for measuring saccadic response times in vision research. Melnik and Pollmann [32] used RELAY to assess how efficient and inefficient visual search tasks influence saccadic re-referencing, while Ganesan et al. [33] utilized it to evaluate gaze-contingent training in individuals with simulated central vision loss. In both cases, RELAY provided a precise and reproducible method for generating artificial saccades, allowing for accurate response time measurements. Additionally, since RELAY is based on an Arduino system, it can be easily expanded to interface with external devices such as photodiodes or other input–output components, enabling synchronized data acquisition and integration with additional experimental hardware. These studies reinforce RELAY’s reliability as a tool for investigating eye movement dynamics and adaptation under controlled experimental conditions.

The differences found for the pupil sizes in dependency on the gaze positions are partly in accordance with the findings of others [11,34]. Although they are statistically significant for all directions, it is noteworthy that if one calculates the percentage deviation from the mean (2888.94, in the tracker’s system units), the deviations range from −3.120% (right) to +2.830% (left) for the horizontal direction and from −1.984% (up) to +1.016% (down) for the vertical direction.

Assuming that the mean reflects a pupil size of 5 mm (the size of our artificial pupil, stated by the manufacturer [22]), the deviation yields a range of 0.298 mm horizontally and 0.150 mm vertically, which is approximately the thickness of a folded letter. The observed differences in this study are a lot smaller than the ones found by Gagl et al. [11] or Hayes and Petrov [34], who reported changes from +5% to −13%, respectively, up to 14.4%, with an artificial eye.

The greatest measured values are for the left and downward directions. It can be assumed that, given the position of the eye tracker’s camera in regard to the artificial eye, which was slightly left and down from it (a typical eye-tracking setup), the biggest pupil sizes occurred when the pupil was looking directly into the camera, hence appearing more round than if the gaze fell into another direction, where pupil appearance was more elliptic.

For future works, it could be interesting to further investigate the geometric relationship between the eye, the tracker and the gaze direction with a desktop mount that puts the lens of the tracker perpendicular to the eye, where the pupil shape should appear really round, and measure the pupil deviations that occur in relation to gaze direction in this setting. This could be used for a comparison between both settings and could lead to the development of a correction algorithm, similar to the approaches of Gagl et al. or Hayes and Petrov [11,34]. If a correction algorithm for the artificial eye is found, one could work on a correction with human participants.

Although small differences were observed in the measured pupil sizes, the mean peak velocities did not change. Thus, it is questionable how big the influence of gaze position really is. A greater problem for estimating could be the distortion of the pupil that happens during saccades with real eyes and leads to the post-saccadic wobble. This is a factor that could not be tested with the rigid eye in this approach but would be worth testing. The relationship between pupil size and gaze position seems to be bidirectional, as pupil size can also influence the measured gaze position [3,35]. In this study, there was only one possible direction of influence because of the fixed pupil. It would be of interest to test with a variable pupil at differing and unchanged positions to find out if both influences have the same weight.

The second objective of this study was to determine whether ambient illumination affects the measurement of saccadic peak velocities and pupil sizes when the pupil size is fixed, specifically investigating whether the eye tracker introduces an artifact under different brightness conditions. Our results suggest that this is not the case. This implies that the Brightness-Induced Roof Pattern (BIRP) in saccadic peak velocities observed in our initial study [2] is likely caused by an unknown human factor rather than a tracking artifact.

The linear relationship between pupil size and brightness cannot explain the observed non-linear peak velocity changes, suggesting an alternative explanation. One possibility is that variations in pupil size affect the estimation of the pupil center, potentially leading to measurement errors in amplitude and peak velocity. However, an influence from rapid pupil size changes seems unlikely, as participants had ample time to adapt to each brightness condition. Additionally, since the mental workload remained consistent during the VGS tasks, it is unlikely that cognitive factors contributed to pupil size variation.

Hessels et al. (2018) [9] argue that the apparent slowing of saccades in the dark is an artifact of pupil-based eye-tracking techniques: “For example, if one were to take ‘saccades’ as ‘detected’ by an algorithm to be true saccades, one might draw wrong conclusions, e.g., that saccades in the dark are slower than saccades in the light. If, on the other hand, one considers the feature which is measured from the eye by the eye tracker, in this case the pupil, it turns out that the apparent slowing of saccades in the dark is an artefact of the eye-tracking technique based on said feature”. Their claim is based on the assumption that eye-tracking algorithms incorrectly detect saccades due to pupil-based tracking errors. However, our results suggest otherwise: Our setup did not exhibit such pupil-dependent changes in peak velocity. Both the peak velocity and the desired coordinates of saccades were detected precisely. This indicates that, at least for the EyeLink 1000 in centroid mode, brightness does not directly induce tracking artifacts in peak velocity measurements. Instead, if brightness-dependent differences are observed in human data, they are more likely to originate from human physiological or cognitive factors rather than a fundamental limitation of the eye-tracking method itself.

Another possible approach for the future could be to conduct a new study with an improved RELAY system (also reduced backlash from dual-axis hinge and using higher resolution motors), where the size of the measured pupil can be adapted to see if they produce a difference in measured peak velocities or not. Depending on the answer to these questions, it would be possible to determine whether an update of the event detection process regarding the surrounding brightness needs to be developed.

As expected, the IR-based tracking was unaffected by ambient brightness. Modern eye trackers, like the EyeLink 1000, use IR illumination to track the pupil, ensuring high contrast regardless of visible light changes. While concerns about BIRP suggested a tracking artifact, our results confirm that changes in saccadic peak velocities stem from human factors, not tracking limitations. However, subtle interactions between gaze position and pupil shape may still introduce minor biases, warranting further investigation. Given our results (including our previous study), we still strongly advise researchers to document the surrounding brightness conditions used in their experiments when possible. Furthermore, we can say that the gaze position while measuring should always be taken into consideration preferably also documented. Maybe a correction algorithm for this can be developed in future approaches. But this would be highly dependent on the used setup geometrics and thus not easy to do.

The MATLAB code for the experiments and additional result presentation can be found in the appendix repository [25]. The software and construction details about RELAY can be found in the GitHub repository [23], which enables an easy reproduction of the low-cost eye robot.

## Figures and Tables

**Table 1:** Table with RGB, Luminance, and Weber contrast $C_{W}$ values for different conditions.

Condition	Dark		Medium		Light	
	**Background**	**Target**	**Background**	**Target**	**Background**	**Target**
R	20	0	100	43	240	95
G	20	0	100	43	240	95
B	20	0	100	43	240	95
Luminance (cd/m²)	2.74	0.89	47.09	6.83	232.03	40.24
$C_{W}$	0.68		0.85		0.83	

**Table 2:** Accuracy for the 13-point calibration grid. Planned Coordinates (τ) are the predefined calibration target positions (true value), Mean Coordinates ($\bar{x}$) represents the average measured gaze position, and Absolute Deviation from τ indicates the offset between planned and measured values.

Metric	Units	X Coordinates												
Planned Coordinates τ	[px]	960.0	960.0	960.0	115.0	1805.0	115.0	1805.0	115.0	1805.0	538.0	1382.0	538.0	1382.0
Mean Coordinates $\bar{x}$	[px]	969.7	979.1	976.6	120.5	1802.1	134.5	1814.0	130.2	1843.5	529.8	1376.7	524.7	1378.8
Absolute Deviation from τ	[px]	9.7	19.1	16.6	5.5	−2.9	19.5	9.0	15.2	38.5	−8.2	−5.3	−13.3	−3.2
Absolute Deviation from τ	[mm]	2.69	5.28	4.60	1.53	−0.81	5.41	2.50	4.21	10.67	−2.27	−1.46	−3.67	−0.88
Absolute Deviation from τ	[°]	0.17	0.33	0.28	0.09	−0.05	0.33	0.15	0.26	0.66	−0.14	−0.09	−0.23	−0.05
	**Units**	**Y Coordinates**												
Planned Coordinates τ	[px]	540.0	92.0	988.0	540.0	540.0	92.0	92.0	988.0	988.0	316.0	316.0	764.0	764.0
Mean Coordinates $\bar{x}$	[px]	532.4	102.1	991.2	546.8	537.7	89.9	79.5	982.4	1005.4	297.5	306.8	753.8	755.1
Absolute Deviation from τ	[px]	−7.60	10.09	3.20	6.85	−2.34	−2.06	−12.5	−5.59	17.43	−18.5	−9.18	−10.2	−8.91
Absolute Deviation from τ	[mm]	−2.10	2.79	0.89	1.90	−0.65	−0.57	−3.45	−1.55	4.82	−5.12	−2.54	−2.83	−2.47
Absolute Deviation from τ	[°]	−0.13	0.17	0.05	0.12	−0.04	−0.04	−0.21	−0.10	0.30	−0.32	−0.16	−0.17	−0.15

**Table 3:** Precision for the 13-point calibration grid. Planned Coordinates (τ) are the predefined calibration target positions (true value), Mean Coordinates ($\bar{x}$) represents the average measured gaze position, and Absolute Deviation from τ indicates the offset between planned and measured values.

Metric	Units	X Coordinates												
Planned Coordinates τ	[px]	960.0	960.0	960.0	115.0	1805.0	115.0	1805.0	115.0	1805.0	538.0	1382.0	538.0	1382.0
Mean Coordinates $\bar{x}$	[px]	969.7	979.1	976.6	120.5	1802.1	134.5	1814.0	130.2	1843.5	529.8	1376.7	524.7	1378.8
Standard Deviation of $\bar{x}$	[px]	0.89	0.88	0.38	0.78	2.24	0.91	0.97	1.26	0.60	2.06	0.28	0.70	0.62
Standard Deviation of $\bar{x}$	[mm]	0.25	0.24	0.11	0.22	0.62	0.25	0.27	0.35	0.17	0.57	0.08	0.19	0.17
Standard Deviation of $\bar{x}$	[°]	0.02	0.02	0.01	0.01	0.04	0.02	0.02	0.02	0.01	0.04	0.00	0.01	0.01
	**Units**	**Y Coordinates**												
Planned Coordinates τ	[px]	540.0	92.0	988.0	540.0	540.0	92.0	92.0	988.0	988.0	316.0	316.0	764.0	764.0
Mean Coordinates $\bar{x}$	[px]	532.4	102.1	991.2	546.8	537.7	89.9	79.5	982.4	1005.4	297.5	306.8	753.8	755.1
Standard Deviation of $\bar{x}$	[px]	0.06	0.75	1.12	0.96	1.76	0.88	0.97	1.58	2.34	2.55	1.03	0.75	0.77
Standard Deviation of $\bar{x}$	[mm]	0.29	0.21	0.31	0.27	0.49	0.24	0.27	0.44	0.65	0.71	0.29	0.21	0.21
Standard Deviation of $\bar{x}$	[°]	0.02	0.01	0.02	0.02	0.03	0.02	0.02	0.03	0.04	0.04	0.02	0.01	0.01

## Data Availability

The original contributions presented in the study are included in the article, further inquiries can be directed to the corresponding author.

## Supplementary Materials

Code for experimental study is openly available in OSF at https://osf.io/xvj62 (accessed on 14 February 2025). The RELAY Project with code and parts list can be found on Github https://github.com/DoStraTech/RELAY (accessed on 14 February 2025).
